# The combined application of antibiotic-loaded bone cement and vacuum sealing drainage for sternal reconstruction in the treatment of deep sternal wound infection

**DOI:** 10.1186/s13019-022-01951-2

**Published:** 2022-08-26

**Authors:** Xia Jiang, Yong Xu, Guoqing Jiao, Zhaohui Jing, Fanyu Bu, Jie Zhang, Liuyan Wei, Xiaosong Rong, Mingqiu Li

**Affiliations:** 1 Department of Cardiovascular Surgery, Wuxi People’s Hospital/The Affiliated Wuxi People’s Hospital of Nanjing Medical University, No. 299 Qingyang Road, Wuxi, 214203 Jiangsu Province China; 2 Department of Chronic Wound, Wuxi Ninth People’s Hospital Affiliated to Soochow University, Wuxi, 214062 China

**Keywords:** Deep sternal wound infection, Antibiotic-loaded bone cement, Vacuum sealing drainage

## Abstract

**Background:**

Deep sternal wound infection (DSWI) is a rare but serious complication after median sternotomy, and treatment success depends mainly on surgical experience. Traditional treatment methods for DSWI include complete debridement, vacuum sealing drainage wound therapy and sometimes transposition of muscle flap. This study aimed to evaluate the utility of antibiotic-loaded bone cement combined with vacuum sealing drainage on DSWI and explore the effect of this treatment on lung function.

**Methods:**

Between January 2018 and December 2019, we treated 12 patients suffering a mediastinitis and open thorax using antibiotic-loaded bone cement combined with vacuum sealing drainage. Subsequently, the blood and local concentration of antibiotic were measured. The patient characteristics, pulmonary function, were retrospectively analyzed. Subjects were followed up for 12 months.

**Results:**

There were no intraoperative deaths. All patients’ healing wounds were first-stage healing without complications and reoperation, the mean hospital stay was 20.2 ± 3.5 days. Local vancomycin concentrations largely exceeded the ones needed for their efficacy while little antibiotic was found in the blood. Pulmonary function testing was improved 2 weeks after the operation. No infection reoccurred in12-month follow-up.

**Conclusions:**

The antibiotic-loaded bone cement combined with vacuum sealing drainage might be an effective method for the sternal reconstruction of deep sternal wound infection and it can improve the patient's lung function in a short time.

## Background

Deep sternal wound infection (DSWI) following cardiac surgery is a devastating complication with the need for subsequent surgical procedures, prolonged hospital stay and increased morbidity and mortality [1–3]. Its incidence is reported between 0.8 and 6.0% and complications are associated with a significant mortality between 1.1 and 19% depending on the literature [4–6].

Conservative treatment methods for DSWI include repeated debridement, occlusive continuous irrigation, and vacuum-assisted closure [7, 8]. However, while these methods are effective to some extent, they often fail to cure patients. Extensive sternal debridement and sternectomy with subsequent muscle or omental flap plasty are often performed in patients treated by plastic surgeons [9]. The recommended approach for DSWI is early resection of the necrotic and infected tissues, vacuum-assisted closure, sternum fixation, and incision closure using various flaps [8, 10]. Despite this, DSWI remains a serious problem, usually necessitating long-term hospitalisation in order for the defect to heal and for a definitive solution to be found, which has dire consequences both for the well-being of the patient and for the economy of the cardiac surgery department.

Antibiotic-loaded bone cement (ALBC) is introduced by Buchholz and Engelbrecht in 1970 [11]. Currently, Polymethylmethacrylate (PMMA) bone cement is an organic polymer material. Because of its good mechanical properties, operating performance and biological inertness, it is widely used for defect filling, fracture stabilization, and arthroplasty [12–14]. Meanwhile, it is the most widely used for loading antibiotics and represents the current standard as an antibiotic delivery vehicle in orthopaedic surgery [15]. But, there are very few reports in the literature that describe the use of the ALBC for DSWI. ALBC can serve as a spacer and as a delivery vehicle for antibiotics, and it can be placed to eliminate dead space.

In this review, we describes our method in treating DSWI after cardiac surgery combined ALBC with VSD and report the preliminary clinical results.

## Methods

### Clinical data

The retrospective study was approved by the hospital ethics committee and informed consent of patients. From January 2018 to December 2019, 12 patients with DSWI following cardiac surgery underwent ALBC and VSD treatment in the department of cardiac surgery at affiliated Wuxi People's Hospital of Nanjing Medical University. In each case, after the diagnosis of DSWI was established, the basic principle of treatment were debridement, administration of culture-specific or broad-spectrum antibiotic. The patient characteristics, pathogenic data, the mean hospital stays were retrospectively analyzed (Table 1).

**Table 1:** Patient characteristics

Characteristics	Value
Total no. of patients (male/female)	8/4
Mean age [years]	62 ± 7.2
Previous medical history	
COPD	3
Diabetes mellitus	3
History of myocardial infarction	2
BMI (kg/m^2^)	25.6 ± 3.2
Type of cardiac surgery	
CABG	4
Valves	4
CABG + valves	2
Aortic operation	2
Internal thoracic artery grafting	3
Re-exploration for bleeding	2
Microorganisms	
GPC	6
GNB	3
Negative	3
Average hospitalization time (days)	20.2 ± 3.5

GNB, gram-negative bacteria; GPC, gram-positive bacteria; Average hospitalization time, hospital stays from antibiotic-loaded bone cement combined with vacuum sealing drainage treatment to discharge; COPD, chronic obstructive pulmonary disease

### Definitions of DSWI

As defined by the Centers for Disease Control and Prevention, DSWI diagnosis requires at least one of the following criteria: (I) an organism is isolated from culture of mediastinal tissue or fluid; (II) evidence of mediastinitis seen during surgery; (III) one of the following conditions: chest pain, sternal instability, or fever (> 38 °C) in combination with either purulent discharge from the mediastinum or isolation of an organism from culture of blood or mediastinal drainage [16].

### Study procedures

We assembled a multidisciplinary team in our department that included cardiac surgeons, chronic wound control physician, Clinical pharmacists, dietitian and nurses.

**Fig. 1:**
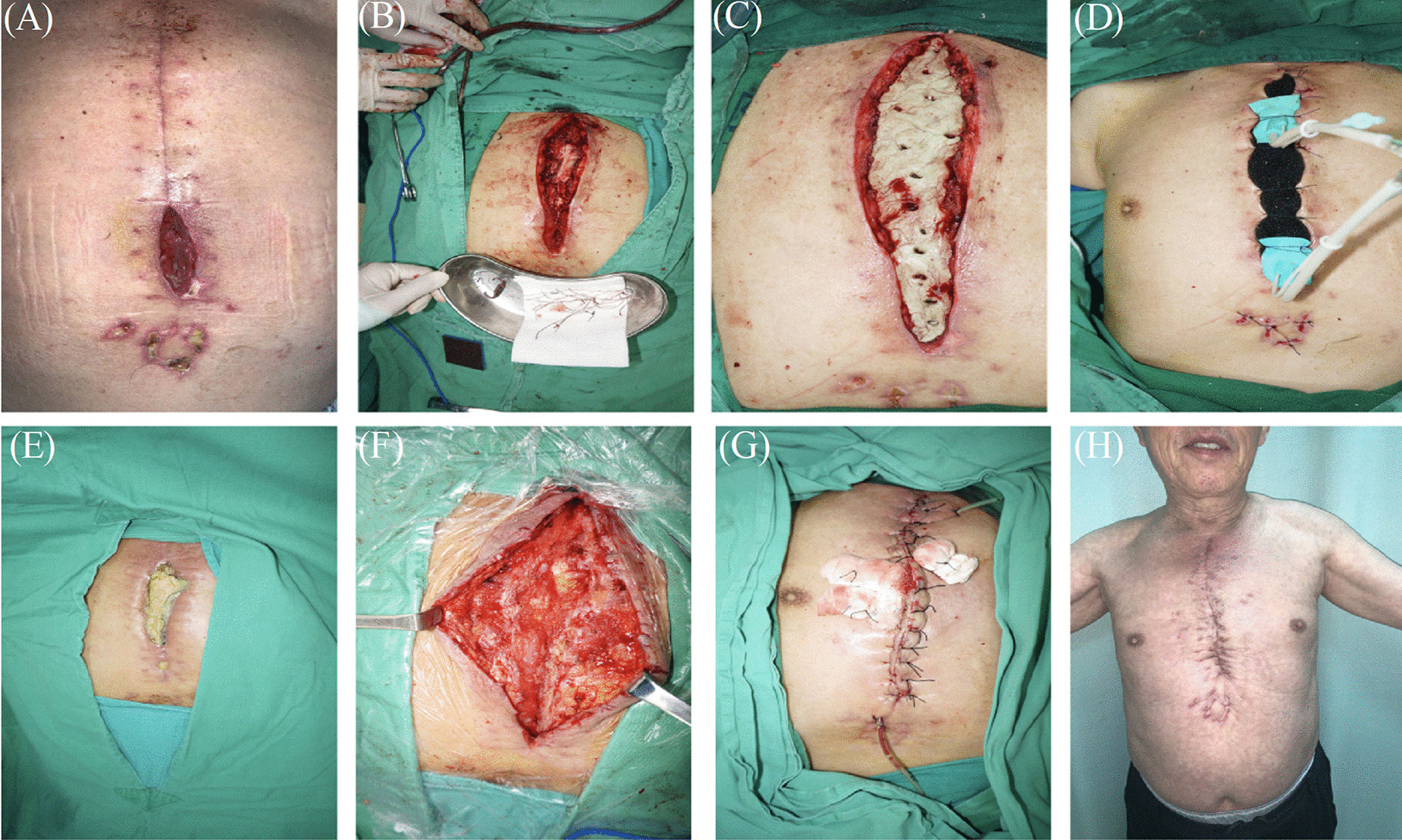
**A** Post-sternotomy wound infection. **B** The wound after debridement. **C** Antibiotic-loaded bone cement covering the sternal defect. **D** VSD was applied over the bone cement. **E** The incision 6 weeks after operation. **F** The bone cement was removed from the incision. **G** The skin and subcutaneous tissue was sutured by methods of relieving tension. **H** Clinical photograph with healed sternal wound

**Fig. 2:**
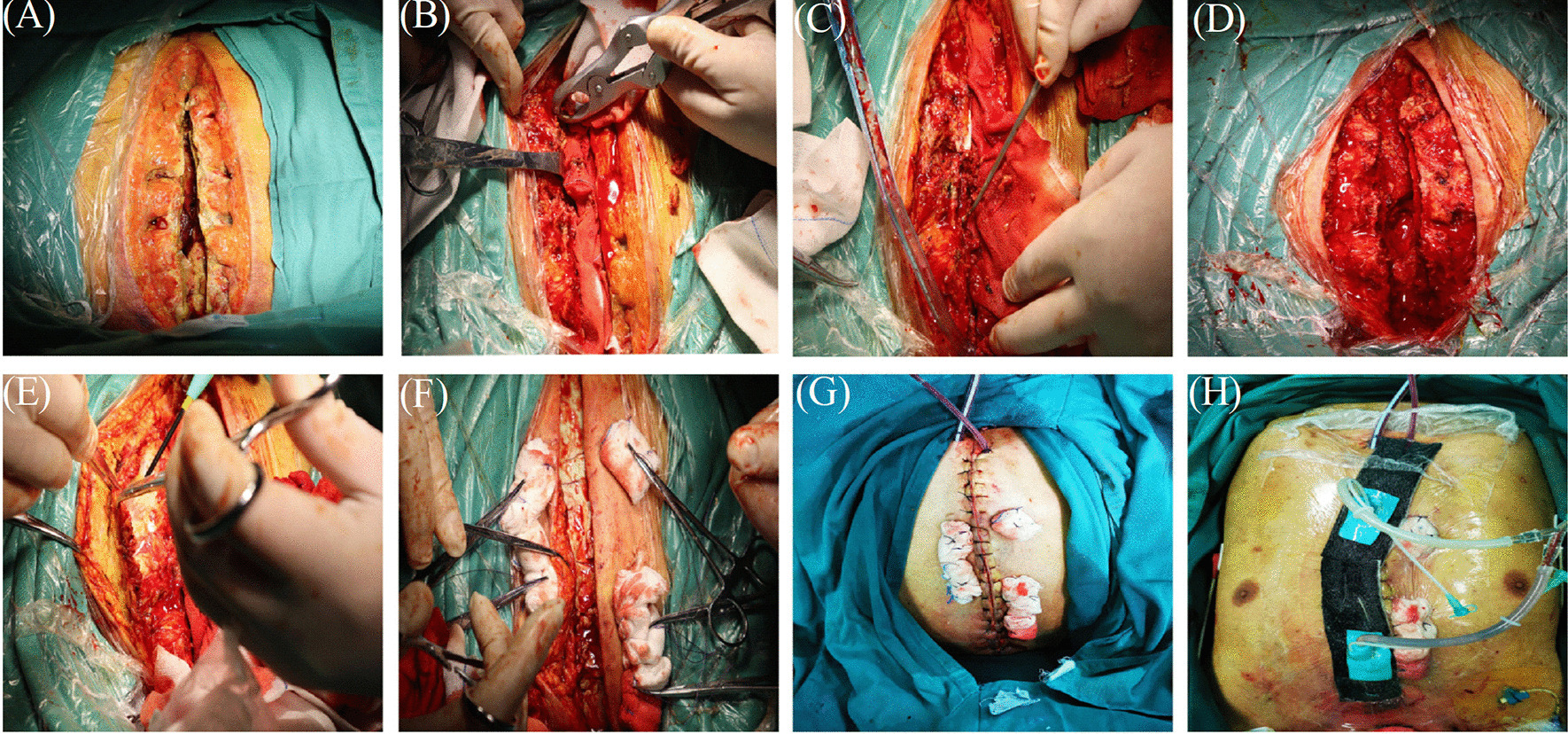
**A** Diagnosis of deep sternal wound infection. **B**–**D** Extensive wound debridement until healthy solid bone with bleeding margins was found. **E** The bilateral pectoralis major muscles were separated from the sternum and subcutaneous. **F** Antibiotic-loaded bone cement covering the sternal defect. **G** The skin was relaxedly sutured without significant tension. **H** The wound with the vacuum sealing drainage dressing in situ

Complete debridement of the wound always took place in the operating room under general anesthesia. A thorough mediastinal wash out was the first step of sternal reconstruction. Next, Operative debridement of infected bone and soft tissue was used, including removal of sternal wires and other foreign bodies (e.g., sutures, pericardial patches, pacemakers) [17]. Then, normal bone tissue with a good blood supply was reached (Figs. 1B, 2B–D). Cultures were also taken from the wound to identify the causative bacteria and adjust the administered antibiotics determined by antibiogram. Next, selection of surgical procedure mainly depends on detection of sternal exposure, sternal instability, extent of sternal bone loss.

Method 1. If infected sternal with minor bone loss (Fig. 1A, B), the ALBC was covered the defect of sternal to provide a reliable bone stability (Fig. 1C). The commercial cements were manufactured with antibiotics premixed, hand-mixed preparations were still commonplace. The amount of gentamycin in this commercial cements (PALACOS MV®+G bone cement, Heraeus, Heraeus Medical GmbH, Wehrheim, Germany) is 0.55 g. The antibiotic loaded cement is prepared by combining a 40 g bag of cement with 2 g of vancomycin or 3.2 g gentamicin powder. The ALBC was placed in the gap between the sternal halves and covered the defect of sternal surface to fix the thorax. Also, the holes were made on the surface of ALBC for drainage (Fig. 1C). The polymerization of bone cement increases the temperature of the cement mixture to 60–80 °C. After bone cement covered the wound completely, the surgeon made holes on the surface of ALBC for drainage with vascular clamp and washed the wound with normal saline to reduce the local temperature. VSD (Wu han VSD Medical Science & Technology Co., Ltd. Vacuum Sealing Drainage Dressing) coverage was provided for the ALBC (Fig. 1D). It was removed when output was less than 5 mL/day for 3 days. When inflammatory markers include white blood cell, ESR, CRP level were normal for a week, the second re-exploration revealed a clean, red, granulating wound bed was achieved (Fig. 1E, F). Two drain tubes (Disposable negative pressure drainage pipeline, AY-Y18-Q400, Ai Yuan medical technology, Jiangsu, China) were placed under the subcutaneous layer. The subcutaneous tissues were intermittently sutured (Fig. 1G). Seven weeks later, the wound looked healthy and showed no evidence of dehiscence or infection (Fig. 1H).

Method 2. If infected sternal with major bone loss (Fig. 2A), pectoral muscle and subcutaneous tissue were mobilized from the chest wall (Fig. 2E)*.* The ALBC was placed in between both sternal halves and covered the defect of sternal to fix the thorax (Fig. 2F). The treatment method of bone cement was the same as method 1. Two drain tubes were placed: one under the muscle flap and the others under the subcutaneous layer. The skin was relaxedly sutured without significant tension (Fig. 2G)*.* Finally, VSD coverage was provided for the wound (Fig. 2H). Connected to the vacuum device, the negative pressure is − 75 to − 100 mmHg. It was removed after intermittent use for about 1 week.

The perioperative management included: maintenance of hemodynamic stability, nutritional support, surveillance and control of perioperative glycemia (< 200 mg/dL), perioperative antibiotic choice, minimization of blood product usage, postoperative wound care, and patient education regarding prevention of incisional infection [18].

### Pulmonary function

Measurements of pulmonary function (n = 12) using volume displacement body plethysmography were carried out by comparing the results to preoperative reference value.

### Blood/local concentration of vancomycin test

The blood concentration of vancomycin (n = 9) was assayed at postoperative day 1, day 3, day 5, day 7, day 9, day 11, day 14 and local concentration was assayed at postoperative day 1, day 3, day 5, day 7.

### Statistical analysis

Continuous variables were expressed as mean ± standard deviation. A paired *t*-test was performed to evaluate the changes in pulmonary function values. A *P* value below 0.05 was considered statistically significant.

## Results

3 patients were treated with Method 1 and 9 patients were treated with Method 2. All patients’ healing wounds were first-stage healing without complications and reoperation, the mean hospitalization time was 20.2 ± 3.5 days. The local concentration of vancomycin remained high throughout the sampling interval with the concentration of (312 ± 73)–(162 ± 36) μg/mL. The blood concentration of vancomycin after ALBC treatment was measured at far below its toxic concentration (60 mg/L) (Table 2), and the function of kidney was all normal during hospitalization. Pulmonary function testing was improved 2 weeks after the operation (*P* < 0.05) (Table 3). No infection reoccurred in12-month follow-up.

**Table 2:** The result of blood/local concentration of vancomycin test (Mean ± SD, μg/mL, n = 9)

Characteristics	BC	LC
Day 1	3.1 ± 0.8	293 ± 93
Day 3	3.0 ± 1.2	312 ± 73
Day 5	2.5 ± 1.3	235 ± 61
Day 7	2.8 ± 0.3	162 ± 36
Day 9	< 2.0	–
Day 11	< 2.0	–
Day 14	< 2.0	–

SD, standard deviation; BC, blood concentration; LC, local concentration

**Table 3:** Pulmonary function tests before bone cement combined with vacuum sealing drainage treatment and at 2 weeks after surgery(n = 12)

Variables	Preoperation	POW 2	*P* value
FVC, mL	2712 ± 650	3265 ± 528	0.02
FVC%	73.2 ± 5.3	85.7 ± 8.2	< 0.01
FEV1, mL	1980 ± 613	2322 ± 520	0.01
FEV1%	70.8 ± 6.5	86.8 ± 8.5	< 0.01
FEV1/FVC, %	65.0 ± 10.7	78.4 ± 10.1	< 0.01

POW, postoperative week; VC, vital capacity; FEV_1_, forced expiratory volume in 1 s; FVC, forced vital capacity

## Discussion

DSWI following cardiac surgery is a serious and dreaded complication. Risk factors for the development of DSWI after median sternotomy include diabetes, obesity, renal dysfunction, smoking history, chronic obstructive pulmonary disease (COPD), use of bilateral internal thoracic artery [19]. Treatment options of DSWI include multiple debridements of necrotic tissues, continuous antibiotherapy, coverage of the defect with well-vascularized tissues such as bilateral pectorals or abdominis rectus muscle flaps or an omentoplasty [9]. Nevertheless, it should be considered as the last step in the treatment of DSWI. Thorough debridement, culture-directed antibiotics and the use of vacuum-assisted therapy (VAC) or VSD significantly decreased mortality and morbidity [1, 20]. The VSD has proven to be the best bridge to reconstruction, preparing the wound until wound closure [9]. In this study, the VSD played the role of adequate drainage. Meanwhile, after suturing derma, VSD device was placed surrounding widely to reduce notch tension, stabilize the thorax and enhance adhesion of the flap to the ALBC.

It is well known that sternal instability triggers mediastinitis and vice versa. So when dealing with DSWI, both the instability and infection must be treated, due to the severe systemic repercussions of both factors [21]. DSWI could often lead to major defects of the anterior chest wall, and it could expose the heart, vessels or any vascular prostheses and coronary grafts [22]. Also, Changes in the properties of the chest wall are likely to directly affect lung volumes and respiratory function. The conventional ALBC is polymethyl methacrylate (PMMA). As PMMA carrier is clinically inert, it does not trigger the host immune response and is able to release antibiotic steadily [23]. Mechanical stability is an important factor to be considered because bone cement has good mechanical properties and is likely to fix infected sternal. It can be adjusted to fill the wound cavity according to the size of the wound defect, leaving no dead space. Mean while, none of the patients complain about residual pain or breathe discomfort due to the stability of sternal. An important point during the ALBC treatment is respiratory function. Patients with muscle flaps surgery may have the persistence of an impaired lung function [24]. In our study, pulmonary function test reached a stable level 2 weeks after surgery (Table 3).

**Fig. 3:**
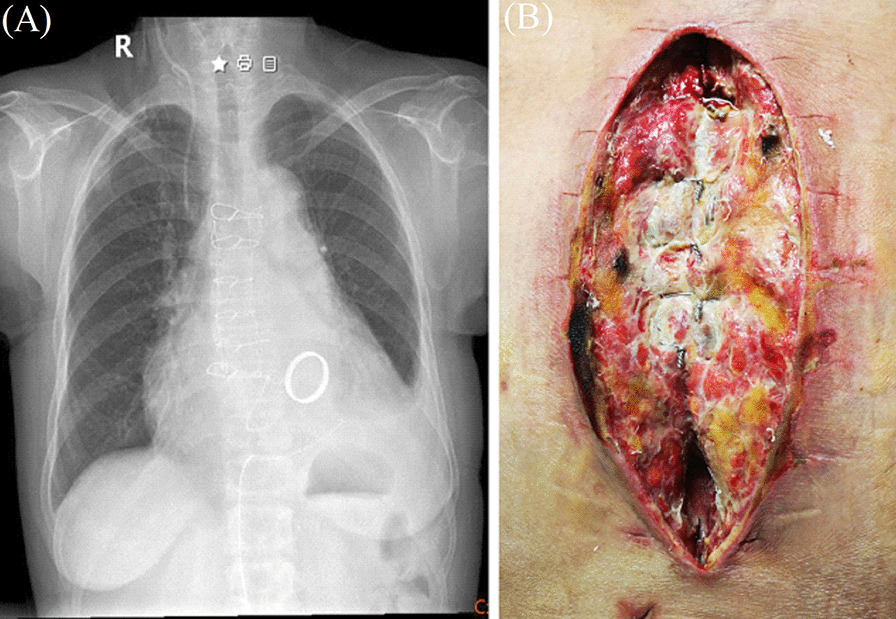
**A** Chest X-ray images of Robiseck sternum closure techniques. **B** Soft tissue ischemia after robiseck closure

Patients with DSWI are typically treated with intravenous antibiotic agents for a few days based on the bacterial culture and drug sensitivity test. However, these systemic modes of administration often achieve only low local (target) drug concentrations due to tissue ischemia, resulting in a minimized local effect. This lack of effect could be due to relatively poor penetration of antibiotic into the bones [25], especially for bilateral internal mammary artery grafts. For some DSWI patients who underwent sternal dehiscence without infection, the Robicsek technique is adopted. However, the success of this fast and cheap method is limited by the quality of the sternal and its disadvantage is that blood supply to the sternal can be disrupted due to wire strangulation around the costal cartilage (Fig. 3). The lack of blood supply to the infectious area limits the amount of antibiotic that can reach the infection site, potentially decreasing the antibacterial effect. Staphylococci, including methicillin-resistant *Staphylococcus aureus* (MRSA), are the most common etiological factors for DSWI [26, 27]. Vancomycin is a member of the glycopeptide class that prevents/destroys several Gram-positive microorganisms, which are the most common pathogens. To target other bacteria and extend the spectrum of efficacy, vancomycin can also be loaded alone or in combination with other antibiotics. Gentamicin is a member of the aminoglycosides class and has a broad spectrum activity. In our study, we chosen vancomycin-loaded bone cement for GPC/Negative-DSWI and gentamicin-loaded bone cement for GNB-DSWI. However, local application of antibiotics can not instead of systemic antibiotics therapy. The antimicrobial treatment was continued intravenously until the clinical picture and serum infected markers erythrocyte sedimentation rate, creactive protein levels showed no signs of infection. In our opinion, the use of antibiotic-loaded bone cement and VSD, in association with a systemic therapy such as antibiotic therapy, nutritional support therapy, anti-coagulant therapy, are the keystone of treatment for DSWI.

Owing to the obvious systemic toxicity by systemic administration, local application of vancomycin is increasingly being adopted to decrease the toxic side effects [28]. However, in the past decade there have been a number of literature reports involving patients who received ALBC during a total knee or hip arthroplasty and subsequently experienced acute renal failure (ARF) [29–31]. Therefore, Safe dose range of local antibiotic administration is important issue for consideration. According to the preoperative drug sensitivity results, intravenous non-vancomycin antibiotics were used in patients with vancomycin positive cultures and negative cultures. The systemic vancomycin dose must be monitored by frequent blood examination. Non-high concentration of vancomycin in the blood guarantees the absence of toxicity (Table 2), indicating that bone cement is safe and effective as a local carrier of vancomycin. The antibiotics that can be used in bone cement preparation are various, in accordance with the particular sensitivity sought. Antibiotics often used in clinical mixed practice are gentamycin vancomycin and tobramycin [32]. The primary advantages of ALBC over conventional intravenous antibiotic delivery are the higher local drug concentration and minimized systemic side effects. During the whole course of treatments, no patients showed obvious renal abnormalities or other complications such as poor wound healing, deep vein thrombosis, pulmonary embolism, or cardio-cerebral vascular accident related to bone cement. The advantages of ALBC combined with VSD method were as follows. (I) It provided enough effective materials to fill the defect and fix the thorax. The stable thorax allowed the patient to achieve full mobility, dietary intake, and rehabilitation after eventual wound closure, thus preserving the lung function. It could also be removed without difficulty if needed. (II) Bone cement was used as a carrier in order to provide high local antibiotic concentrations, with lower incidence of adverse effects related to antibiotic therapy, lower risk of creating antibiotic resistance. (III) A radical debridement of the infected and necrotic soft tissue and bone material should be carried out until a well-perfused and vascularized tissue had been exposed. Therefore, partial resection of the sternal had to be performed. (IV) High suture tension is one of the causes for many wound-healing problems. The new suture technique was used to fix the muscle flap and skin, which reduced the occurrence of foreign-body infections.

## Conclusions

The prevalence of midline sternotomy as the most commonly used surgical approach in open heart surgery has resulted in post-sternotomy deep wound infections remaining a major challenge. The management of DSWI requires early and sufficient surgical debridement, combined with bactericidal antibiotic therapy. Herein, we described a DSWI protocol involving implantation of antibiotic-loaded bone cement combined with VSD because of its excellent mechanical properties and sustainable drug delivery. Future studies will be conducted to determine if this technique could be used as a treatment strategy for DSWI.

### Limitations

Many questions, including what is the optimal antibiotic dose, What kind of patient would benefit from it and which is the optimal antibiotic-cement combination to eradicate microorganisms specifically, are still open. This is a retrospective case series presentation with no comparison group, the sample size is small, and the follow-up period is short. It remains to be seen whether there are related complications caused by ALBC in the long-term follow-up.

## Data Availability

The datasets generated and analyzed during the current study are not published due to the use of internal records of patient data and established privacy policies, but will be available from the corresponding author upon reasonable request.

## Abbreviations

ALBC: Antibiotic-loaded bone cement
VSD: Vacuum sealing drainage
ESR: Erythrocyte sedimentation rate
CRP: C-reactive protein
